# Draft Genome Sequence of Freshwater-Derived *Streptomyces* sp. Strain BPSDS2, Isolated from Damte Stream, Northeast India

**DOI:** 10.1128/MRA.00874-19

**Published:** 2019-10-24

**Authors:** Zothanpuia, Ajit Kumar Passari, Purbajyoti Deka, Vinay Rajput, Lakshmi P. M. Priya, Mahesh Dharne, Syed Dastager, Oommen K. Mathew, Abeer Hashem, Elsayed Fathi Abd_ Allah, Bhim Pratap Singh

**Affiliations:** a Department of Biotechnology, Mizoram University, Aizawl, Mizoram, India; b NCIM Resource Centre, CSIR-National Chemical Laboratory, Pune, India; c AgriGenome Labs Pvt Ltd., SmartCity Kochi, Kerala, India; d Botany and Microbiology Department, College of Science, King Saud University, Riyadh, Saudi Arabia; e Mycology and Plant Disease Survey Department, Plant Pathology Research Institute, Agriculture Research Center, Giza, Egypt; f Plant Production Department, College of Food and Agriculture Science, King Saud University, Riyadh, Saudi Arabia; University of Delaware

## Abstract

We report the draft genome sequence of Streptomyces sp. strain BPSDS2, isolated from freshwater sediments in Northeast India. The draft genome has a size of 8.27 Mb and 7,559 protein-coding sequences.

## ANNOUNCEMENT

Streptomyces spp. are Gram-positive filamentous spore-forming bacteria, belong to the most dominant genus of the *Actinobacteria,* have high G+C content in their DNA (1), and are remarkably rich sources of bioactive compounds, accounting for the production of over two-thirds of the commercially available antibiotics in current use (2). *Streptomyces* spp. account for the production of 75% of antibiotics in use from the total of 70% of antibiotics produced by the phylum *Actinobacteria* (3). In the course of our screening program for new bioactive compounds from freshwater actinobacteria, *Streptomyces* sp. strain BPSDS2 was isolated from a freshwater sediment sample from Damte Stream (23°73′N, 92°80′E) in Mizoram, Northeast India.

The sediment sample was diluted in distilled water, and the dilutions were spread over the surface of starch casein agar (SCA) solid agar medium. The plates were incubated at 28°C for 7 days, and pure colonies were obtained after repeated subculturing on fresh isolation medium (4). The pure colonies were then transferred to tryptone-yeast extract broth (ISP1 broth) liquid medium, and axenicity was confirmed by Gram staining. Cells were harvested, and genomic DNA was extracted using the PureLink genomic DNA isolation kit (catalog number K182002; Thermo Scientific Invitrogen). The 16S rRNA gene was amplified using universal bacterial primers. The primers, reactions, and conditions of the PCR were exactly as reported in our previous studies (5). The obtained sequences were compared with the reference strains of actinobacteria from the NCBI genomic database using a BLASTn search to determine similarity percentages. Based on 16S rRNA gene sequence analysis, strain BPSDS2 (GenBank accession number MG711553) was found to be closely similar to *Streptomyces* sp. strain QLS87 (GenBank accession number JQ838120), with 99% similarity.

For genome sequencing, 500 ng of the isolated good-quality genomic DNA was fragmented using a Covaris M220 sonicator. The fragmented genomic DNA was end repaired and further processed for ligation of Illumina adaptors with the NEBNext Ultra DNA library preparation kit (catalog number E7370L; NEB), as per the recommendation of the manufacturer. An adaptor-ligated enriched library was purified using AMPure XP beads. Library size distribution was checked on an Agilent TapeStation D1000 DNA chip (product number 5067-5583). *Streptomyces* sp. strain BPSDS2 genomic DNA was sequenced with the 2 × 150-bp paired-end read length sequencing protocol of the Illumina MiSeq platform. The quality check of the reads was done using FastQC (6), and the generated sequencing reads were filtered to remove low-quality reads using Trim Galore v0.5.0 (7), with set default parameters. Unicycler v0.4.8 (8) was used for *de novo* assembly. The *Streptomyces* sp. strain BPSDS2 draft genome sequence contains 54 contigs, with an *N*_50_ value of 349,850 bp. The genome coverage is ~234.0×, and the estimated genome length is 8,272,875 bp, with an average G+C content of 71.82%. The *Streptomyces* sp. strain BPSDS2 genome was annotated on the PATRIC Web server (9) using the RAST tool kit (RASTtk) (10), and it contains 7,546 protein-coding sequences (CDS), 65 tRNA genes, and 3 rRNA genes. The annotation of this strain consists of 2,498 hypothetical proteins and 5,048 proteins with functional assignments, including 1,233 proteins with Enzyme Commission (EC) numbers, 1,070 with Gene Ontology (GO) assignments, and 968 proteins that were mapped to the KEGG pathway.

### Data availability.

This whole-genome shotgun project has been deposited at the NCBI database under GenBank accession numbers STGN01000001 to STGN01000054. The BioSample accession number is SAMN11159182. The BioProject identifier is PRJNA527763. The short-read data have been submitted to the SRA under run accession number SRR8742575.
